# Variations in infection sites and mortality rates among patients in intensive care units with severe sepsis and septic shock in Japan

**DOI:** 10.1186/s40560-019-0383-3

**Published:** 2019-05-03

**Authors:** Toshikazu Abe, Hiroshi Ogura, Shigeki Kushimoto, Atsushi Shiraishi, Takehiro Sugiyama, Gautam A. Deshpande, Masatoshi Uchida, Isao Nagata, Daizoh Saitoh, Seitaro Fujishima, Toshihiko Mayumi, Toru Hifumi, Yasukazu Shiino, Taka-aki Nakada, Takehiko Tarui, Yasuhiro Otomo, Kohji Okamoto, Yutaka Umemura, Joji Kotani, Yuichiro Sakamoto, Junichi Sasaki, Shin-ichiro Shiraishi, Kiyotsugu Takuma, Ryosuke Tsuruta, Akiyoshi Hagiwara, Kazuma Yamakawa, Tomohiko Masuno, Naoshi Takeyama, Norio Yamashita, Hiroto Ikeda, Masashi Ueyama, Satoshi Fujimi, Satoshi Gando, on behalf of JAAM FORECAST group, Osamu Tasaki, Yasumitsu Mizobata, Hiraku Funakoshi, Toshiro Okuyama, Iwao Yamashita, Toshio Kanai, Yasuo Yamada, Mayuki Aibiki, Keiji Sato, Susumu Yamashita, Kenichi Yoshida, Shunji Kasaoka, Akihide Kon, Hiroshi Rinka, Hiroshi Kato, Hiroshi Okudera, Eichi Narimatsu, Toshifumi Fujiwara, Manabu Sugita, Yasuo Shichinohe, Hajime Nakae, Ryouji Iiduka, Mitsunobu Nakamura, Yuji Murata, Yoshitake Sato, Hiroyasu Ishikura, Yasuhiro Myojo, Yasuyuki Tsujita, Kosaku Kinoshita, Hiroyuki Yamaguchi, Toshihiro Sakurai, Satoru Miyatake, Takao Saotome, Susumu Yasuda, Yasuaki Mizushima

**Affiliations:** 1 Department of General Medicine, Juntendo University, 2-1-1 Hongo, Bunkyo-ku, Tokyo, 113-0033 Japan; 2 Health Services Research and Development Center, University of Tsukuba, Tsukuba, Japan; 3 Department of Traumatology and Acute Critical Medicine, Osaka University Graduate School of Medicine, Suita, Japan; 4 Division of Emergency and Critical Care Medicine, Tohoku University Graduate School of Medicine, Sendai, Japan; 5 Emergency and Trauma Center, Kameda Medical Center, Kamogawa, Japan; 6 Diabetes and Metabolism Information Center, Research Institute, National Center for Global Health and Medicine, Tokyo, Japan; 7 Division of Traumatology, Research Institute, National Defense Medical College, Tokorozawa, Japan; 8 Center for General Medicine Education, Keio University School of Medicine, Tokyo, Japan; 9 Department of Emergency Medicine, School of Medicine, University of Occupational and Environmental Health, Kitakyushu, Japan; 10 Department of Emergency and Critical Care Medicine, St. Luke’s International Hospital, Tokyo, Japan; 11 Department of Acute Medicine, Kawasaki Medical School, Kurashiki, Japan; 12 Department of Emergency and Critical Care Medicine, Chiba University Graduate School of Medicine, Chiba, Japan; 13 Department of Trauma and Critical Care Medicine, Kyorin University School of Medicine, Mitaka, Japan; 14 Trauma and Acute Critical Care Center, Medical Hospital, Tokyo Medical and Dental University, Tokyo, Japan; 15 Department of Surgery, Center for Gastroenterology and Liver Disease, Kitakyushu City Yahata Hospital, Kitakyushu, Japan; 16 Department of Disaster and Emergency Medicine, Kobe University Graduate School of Medicine, Kobe, Japan; 17 Emergency and Critical Care Medicine, Saga University Hospital, Saga, Japan; 18 Department of Emergency and Critical Care Medicine, Keio University School of Medicine, Tokyo, Japan; 19 Department of Emergency and Critical Care Medicine, Aizu Chuo Hospital, Aizuwakamatsu, Japan; 20 Emergency & Critical Care Center, Kawasaki Municipal Kawasaki Hospital, Kawasaki, Japan; 21 Advanced Medical Emergency & Critical Care Center, Yamaguchi University Hospital, Ube, Japan; 22 Department of Emergency Medicine, Niizashiki Chuo General Hospital, Niiza, Japan; 23 Division of Trauma and Surgical Critical Care, Osaka General Medical Center, Osaka, Japan; 24 Department of Emergency and Critical Care Medicine, Nippon Medical School, Tokyo, Japan; 25 Advanced Critical Care Center, Aichi Medical University Hospital, Nagakute, Japan; 26 Advanced Emergency Medical Service Center, Kurume University Hospital, Kurume, Japan; 27 Department of Emergency Medicine, Teikyo University School of Medicine, Tokyo, Japan; 28 Department of Trauma, Critical Care Medicine, and Burn Center, Japan Community Healthcare Organization, Chukyo Hospital, Nagoya, Japan; 29 Division of Acute and Critical Care Medicine, Hokkaido University Graduate School of Medicine, Sapporo, Japan; 30 Department of Health Services Research, Faculty of Medicine, University of Tsukuba, Tsukuba, Japan; 31 Department of Acute and Critical Care Medicine, Sapporo Higashi Tokushukai Hospital, Sapporo, Japan

**Keywords:** Heterogeneity, Survival, Therapy, Sepsis, Infection

## Abstract

**Background:**

Accurate and early identification of infection sites might help to drive crucial decisions regarding the treatment of sepsis. We aimed to determine the clinical and etiological features of infection according to sites among patients with severe sepsis in Japan.

**Methods:**

This secondary analysis of a multicenter, prospective cohort study included 59 intensive care units (ICU) and proceeded between January 2016 and March 2017. The study cohort comprised 1184 adults (≥ 16 years) who were admitted to an ICU with severe sepsis and septic shock diagnosed according to the sepsis-2 criteria. Sites of infection diagnosed by physicians in charge at the time of arrival comprised the lung, abdomen, urinary tract, soft tissue, bloodstream, central nervous system (CNS), and undifferentiated infections. The primary outcome was in-hospital mortality.

**Results:**

The most common sites of infection were the lungs (31.0%), followed by intra-abdominal sites (26.3%), the urinary tract (18.4%), and soft tissue (10.9%). The characteristics of the patients with severe sepsis across seven major suspected infection sites were heterogeneous. Septic shock was more frequent among patients with intra-abdominal (72.2%) and urinary tract (70.2%) infections than other sites. The in-hospital mortality rate due to severe sepsis and septic shock of a pooled sample was 23.4% (range, 11.9% [urinary tract infection] to 47.6% [CNS infection]). After adjusting for clinical background, sepsis severity, and stratification according to the presence or absence of shock, variations in hospital mortality across seven major sites of infection remained essentially unchanged from those for crude in-hospital mortality; adjusted in-hospital mortality rates ranged from 7.7% (95%CI, − 0.3 to 15.8) for urinary tract infection without shock to 58.3% (95%CI, 21.0–95.7) for CNS infection with shock in a generalized estimating equation model. Intra-abdominal and urinary tract infections were statistically associated with less in-hospital mortality than pneumonia. Infections of the CNS were statistically associated with higher in-hospital mortality rates than pneumonia in a logistic regression model, but not in the generalized estimating equation model.

**Conclusions:**

In-hospital mortality and clinical features of patients with severe sepsis and septic shock were heterogeneous according to sites of infection.

**Electronic supplementary material:**

The online version of this article (10.1186/s40560-019-0383-3) contains supplementary material, which is available to authorized users.

## Introduction

Sepsis remains a major lethal healthcare problem, with a reported mortality of > 25% [1]. Sepsis differs from straightforward infection in that it is associated with life-threatening organ dysfunction, multiple organ failure, and death due to a dysregulated host response to infection [2]. The pathology of sepsis involves both inflammatory and anti-inflammatory responses [3, 4] and includes a broad spectrum of conditions with various clinical manifestations and patterns of acute organ dysfunction. From a clinical viewpoint, the history and characteristics of patients, infection sites, and comorbidities are remarkably heterogeneous. This, together with confusing nomenclature describing this syndrome and the scarcity of appropriate epidemiological data, has contributed to suboptimal findings from observational studies of sepsis [5–7].

Some authors have suggested that understanding clinical differences based on infection sites could aid clinicians to appropriately stratify risk, help guide clinical decisions regarding treatment, and facilitate understanding of variations in host responses [8, 9]. However, few studies have described differences in clinical characteristics and in-hospital mortality based on infection sites that might be important [6, 7]. Although our previous Focused Outcomes Research in Emergency Care in Acute Respiratory Distress Syndrome, Sepsis, and Trauma (FORECAST) study [10] determined management and clinical outcomes among patients with severe sepsis and septic shock in Japan, clinical features according to infection sites have not yet been evaluated. Therefore, the present study aimed to identify the clinical and etiological features and outcomes of severe sepsis based on infection sites to help guide clinical decisions.

## Methods

### Design, setting, and participants

This is a secondary analysis of a sub-study of patients with severe sepsis in the multicenter prospective cohort FORECAST study of acutely ill patients at 59 ICU in Japan that proceeded between January 2016 and March 2017 [10]. We investigated data from adult patients (≥ 16 years) diagnosed with severe sepsis based on the 2003 sepsis-2 criteria [11]. Inclusion criteria comprised the new onset of suspected infection based on clinical history, at least two systemic inflammatory response syndrome (SIRS) criteria, and evidence of dysfunction in at least one organ [11]. Exclusion criteria comprised requiring sustained life support or being in post-cardiopulmonary arrest resuscitation status when sepsis was diagnosed.

### Data collection

Data were obtained from a database compiled by the FORECAST investigators. The variable of primary interest was the site of infection. Other variables included patient information such as demographics, admission source, comorbidities, activities of daily life (ADL) status, organ dysfunction, infection characteristics, laboratory data, blood culture findings, and antibiotics (no information was available about the amount and duration). Data were inputted by FORECAST investigators throughout the periods that patients remained in hospital. The primary outcome was in-hospital mortality.

### Data definitions

Septic shock and organ dysfunction were defined according to the sepsis-2 criteria [11]. Sites of infection were assessed as suspected at initial examination by physicians in charge because the initial diagnosis contributes to predicting outcome [6, 7]. Sites of infection in this database initially included the lungs, intra-abdominal sites (the peritoneum, the pancreas, the gall bladder, the bowel, and other sites), urinary tract, soft tissue, wounds, osteo-articular sites, endocardium, and catheter-related, implant device-related, central nervous system (CNS), and undifferentiated infection (Additional file 1: Table S1). Because several categories contained few patients, we consolidated them into seven major categories comprising the lungs, intra-abdomen, urinary tract, soft tissue (including wounds), and bloodstream-related, CNS, and undifferentiated infection.

### Analysis

Descriptive statistics included counts (proportions) for categorical variables, and continuous variables are expressed as medians with interquartile ranges (IQR) because many variables were not normally distributed. Data were not adjusted unless specifically stated otherwise. Since few values were missing, assumptions were not made for missing data; these are noted as footnotes to tables.

We compared the baseline characteristics of the patients, demographic data, infection characteristics including results of blood cultures, sepsis severity, organ dysfunction, and mortality outcomes categorized by infection sites. We also described the frequency and choice of initial antibiotics administered. We then stratified crude in-hospital mortality rates by sites of infection and the presence of shock because clinical approaches such as resuscitation differed between patients with and without shock. We adjusted the backgrounds of the patients and sepsis severity considering clustering by ICU using generalized estimating equation (GEE) models with an independent working correlation matrix. Models were adjusted for age, sex, Charlson comorbidity index (CCI), and organ-specific sepsis-related organ failure assessment (SOFA) scores and were selected a priori based on reported findings [6, 7] and clinical importance. We then used marginal standardization [12] based on probability determined from the GEE model to estimate adjusted in-hospital mortality by the seven major infection sites. To determine the clinical relationship between infection sites and shock, these models were further stratified by the presence or absence of shock. Results are reported as adjusted in-hospital mortality rates with 95% confidence intervals (CI). We assessed each variable for multicollinearity and also compared in-hospital mortality rates among the seven major infection sites using logistic regression as a sensitivity analysis. Models were adjusted for age, sex, BMI, ADL, admission sources, Charlson comorbidity index (CCI), presence of shock, and organ-specific SOFA scores, then further stratified by the presence of shock. The lungs served as the reference for all statistical models as they comprised the most frequent sites of infection.

All *p* values were two-sided, with *p* < 0.05 being considered statistically significant. Data were statistically analyzed using SPSS software, version 23.0 (IBM, Armonk, NY, USA) and Stata software, version 14.2 (StataCorp, College Station, TX, USA).

## Results

### Patients’ characteristics

We analyzed data from 1184 patients with severe sepsis who met the inclusion and exclusion criteria at 59 participating ICU in Japan between January 2016 and March 2017. The median age was 73 (IQR, 64–81) years and 60.7% were male. Most (57.1%) patients with severe sepsis arrived directly from emergency departments (ED), fewer were transferred from other departments or were diagnosed with severe sepsis while in an ICU, and 62.9% of patients were diagnosed with septic shock on arrival. The rate of blood culture positivity upon admission was 54.0%.

Patients were categorized by infection site upon arrival (Table 1). The most common site of infection was the lungs (31.0%), followed by the intra-abdomen (26.3%), the urinary tract (18.4%), and soft tissue (10.9%). The characteristics of the patients with severe sepsis were heterogeneous across all seven infection sites. Septic shock was more frequent among patients with intra-abdominal (72.2%) and urinary tract (70.2%) infections. Patients with lung, CNS, and undifferentiated infections had high APACHE II scores. Organ-specific SOFA scores were heterogeneous, but total scores were relatively similar. Rates of blood culture positivity were > 80% in bloodstream-related (osteo-articular 81.0%, endocardial 81.3%, catheter-related 90.9%, and implant device-related 87.5%) infections (Additional file 1: Table S1). Gram-negative rods were most frequently identified in cultured blood from intra-abdominal and urinary tract sites, whereas Gram-positive cocci were prevalent at other sites of infection (Additional file 2: Table S2). Fungal infection was rarely found in blood cultures. With respect to organ dysfunction, hypotension and hyperlactatemia were equally prevalent among patients with shock (Additional file 2: Table S2). Hyperlactatemia was prevalent (69.6%) among patients with CNS infection, although the rate of shock was relatively low (30.4%). The prevalence of thrombocytopenia was 43.5% and 55.1% in CNS and undifferentiated infections, respectively.

**Table 1:** Characteristics of patients with sepsis and major infection sites (*n* = 1184)

Characteristics	All	Sites						
		Lung	Intra-abdomen	Urinary tract	Soft tissue	Bloodstream	CNS	Undifferentiated
	1184	367 (31.0)	311 (26.3)	218 (18.4)	129 (10.9)	67 (5.7)	23 (1.9)	69 (5.8)
Age at admission (years)	73 (64–81)	73 (65–80)	74 (66–83)	74 (63–83)	67 (58–78)	69 (57–81)	69 (63–84)	69 (58–75)
Male sex	719 (60.7)	263 (71.7)	191 (61.4)	94 (43.1)	80 (62.0)	36 (53.7)	15 (65.2)	40 (58.0)
BMI (kg/m^2^)	22 (19–25)	22 (19–25)	21 (19–24)	22 (19–25)	23 (21–26)	22 (19–23)	23 (21–25)	24 (20–25)
ADL (Inactive)	288 (24.3)	82 (22.3)	60 (19.4)	82 (37.6)	29 (22.7)	22 (32.8)	1 (4.3)	12 (17.9)
Charlson comorbidity index	1 (0–2)	2 (0–3)	1 (0–2)	1 (0–2)	1 (0–2)	1 (0–2)	1 (1–2)	1 (1–3)
Admission source								
ED	676 (57.2)	226 (61.6)	158 (51.1)	147 (67.4)	67 (51.9)	32 (47.8)	12 (52.2)	34 (49.3)
Non-ED (transfer or other department)	457 (38.7)	131 (35.7)	134 (43.4)	68 (31.2)	55 (42.6)	31 (46.3)	7 (30.4)	31 (44.9)
ICU	49 (4.1)	10 (2.7)	17 (5.5)	3 (1.4)	7 (5.4)	6 (6.0)	4 (17.4)	4 (5.8)
Septic Shock	745 (62.9)	197 (53.7)	226 (72.2)	153 (70.2)	75 (58.1)	41 (61.2)	7 (30.4)	46 (66.7)
Positive blood culture	636 (54.0)	133 (36.4)	154 (49.8)	160 (73.7)	75 (58.1)	57 (85.1)	16 (72.7)	41 (60.3)
APACHE II score	23 (17–29)	25 (18–31)	21 (16–28)	22 (17–27)	22 (15–28)	21 (17–30)	27 (20–33)	26 (18–34)
SOFA score								
All	9 (6–11)	9 (5–12)	9 (6–11)	9 (6–11)	8 (5–11)	8 (6–11)	9 (7–11)	10 (7–13)
Respiratory	2 (1–2)	2 (2–3)	2 (1–2)	2 (1–2)	2 (1–2)	1 (0.3–2)	1 (1–2)	2 (1–2)
Cardiovascular	3 (0–4)	2 (0–4)	3 (1–4)	3 (0–4)	3 (0–4)	2.5 (0–4)	0 (0–4)	0 (0–3)
Hepatic	0 (0–1)	0 (0–1)	0 (0–2)	0 (0–2)	0 (0–1)	0 (0–1.8)	0 (0–1)	0 (0–2)
Coagulation	1 (0–2)	0 (0–1)	1 (0–1)	1 (0–2)	1 (0–2)	1 (0–2)	1 (0–2)	2 (0–3)
Renal	1 (0–3)	1 (0–3)	1 (0–3)	2 (1–3)	2 (0–3)	2 (0–3)	1 (1–2)	2 (0.3–3.8)
Neurological	1 (0–3)	1.5 (0–3)	1 (0–2)	1 (0–3)	1 (0–2)	1 (0–2)	3 (2–3)	1 (0–3)
Broad spectrum antibiotics								
Carbapenem	627 (55.0)	159 (45.0)	182 (61.3)	127 (59.6)	79 (63.7)	30 (44.8)	10 (45.5)	40 (62.5)
PIPC/TAZ	238 (20.9)	85 (24.1)	59 (19.9)	52 (24.4)	20 (16.1)	16 (23.9)	0 (0)	6 (9)

Reported counts (proportions) of categorical and medians (interquartile range) for continuous variables. Missing data: BMI, *n* = 26; admission source, *n* = 2; APACHE II score, *n* = 16; SOFA score, *n* = 183

*ADL* activities of daily living, *APACHE* acute physiology and chronic health evaluation, *BMI* body mass index, *ED* emergency department, *SIRS* systemic inflammatory response syndrome, *SOFA* sepsis-related organ failure assessment

Carbapenem was the most frequent initial antibiotic (55.0%), followed by tazobactam/piperacillin (PIPC/TAZ) (20.9%) (Table 1). Carbapenem was usually administered to patients with intra-abdominal, urinary tract, and soft tissue infections (61.3%, 59.6%, and 63.7%, respectively), whereas bloodstream and CNS infections were usually treated with vancomycin (VCM; 47.8% and 63.6%, respectively) (Additional file 3: Table S3). Most (63.2%) patients received antibiotic monotherapy, particularly those with intra-abdominal (79.8%) or urinary tract (77.5%) infections, whereas those with soft tissue, bloodstream, and CNS infections more frequently received combined antibiotics (66.9%, 62.7%, and 72.7%, respectively).

### Outcomes

**Fig. 1:**
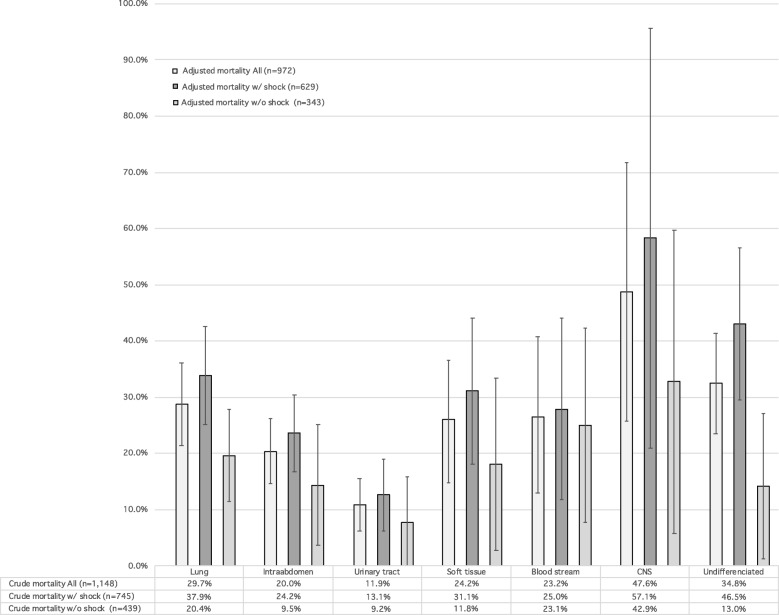
Crude and adjusted in-hospital mortality rates for patients with sepsis according to seven major infection sites stratified by shock. The crude mortalities are summarized in the table and adjusted mortalities and its 95% confidence interval calculated by the generalized estimating equation model are demonstrated with a bar graph with error bars. Data were adjusted for age, sex, Charlson comorbidity index, and organ-specific sepsis-related organ failure assessment (SOFA) scores using marginal standardization. CNS central nervous system

**Fig. 2:**
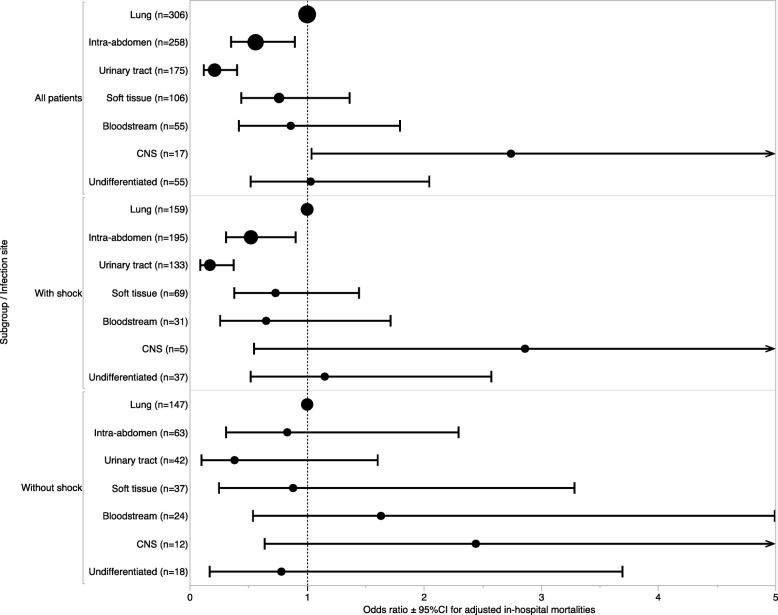
Relationship between seven major sites of infection and in-hospital mortality among all patients with severe sepsis (*n* = 952), and those with (*n* = 613) and without (*n* = 339) septic shock using logistic regression models. Data were adjusted for age, sex, BMI, ADL, admission sources, CCI, presence of shock, and organ-specific SOFA scores. ADL, activities of daily living; BMI, body mass index; CCI, Charlson comorbidity index; CI, confidence interval; CNS, central nervous system; SOFA, sepsis-related organ failure assessment

Mortality follow-up data were available for 97.0% of patients during their hospitalization. The overall in-hospital mortality rate was 23.4%. Regardless of shock status, crude in-hospital mortality ranged from 11.9 to 47.6% (urinary tract infection and CNS infection, respectively) across all sites of infection (Fig. 1; Additional file 1: Table S1). After stratification by the presence or absence of shock, crude in-mortality values ranged from 9.2% (urinary tract infection without shock) to 57.1% (CNS infection with shock) (Fig. 1). After adjusting for clinical background and sepsis severity, variations in hospital mortality across the seven major sites of infection remained essentially unchanged from those for crude in-hospital mortality; adjusted in-hospital mortality ranged from 7.7% (95%CI, − 0.3 to 15.8) for urinary tract infection without shock to 58.3% (95%CI, 21.0–95.7) for CNS infection with shock in a generalized estimating equation model (Fig. 1). Adjusted mortalities and its 95% confidence interval calculated by the generalized estimating equation model are demonstrated; those for pneumonia were 28.8% (95%CI, 21.4–36.2) in all, 33.9% (95%CI, 25.1–42.6) with shock, and 19.6% (95%CI, 11.4–27.8) without shock; those for CNS infection were 48.7% (95%CI, 25.7–71.7) in all, 58.3% (95%CI, 21.0–95.7) with shock, and 32.7% (95%CI, 5.8–59.7) without shock; and those for undifferentiated infection were 32.4% (95%CI, 23.5–41.4) in all, 43.1% (95%CI, 29.6–56.6) with shock, and 14.1% (95%CI, 1.2–27.1) without shock. Intra-abdominal infections were statistically associated with lower in-hospital mortality rates than pneumonia in the logistic regression model (*P* = 0.03) but not in the GEE model (*P* = 0.10). Urinary tract infections were statistically associated with lower in-hospital mortality rates than pneumonia in the logistic model and in the GEE model (*P* < 0.01). Infections of the CNS were statistically associated with higher in-hospital mortality rates than pneumonia in the logistic regression model (*P* = 0.04), but not in the GEE model (*P* = 0.07). Multicollinearity was not observed in the GEE model and the logistic regression model; all variance inflation factors were less than 3. After stratification by shock, variations in in-hospital mortality according to sites of infection persisted in patients with septic shock but not in those with non-septic shock (Figs. 1 and 2).

## Discussion

### Brief summary

The multicenter, prospective FORECAST cohort study of patients with severe sepsis at 59 ICU in Japan confirmed that severe sepsis is a heterogeneous clinical syndrome that is associated with a poor prognosis. More importantly, highly variable in-hospital mortality was associated with infection sites, especially among patients with shock.

### Heterogeneity of severe sepsis

The variation in severe sepsis was associated with infection sites and shock. After adjustment for initial characteristics and severity, intra-abdominal or urinary tract infections were associated with lower mortality rates than other sites of infection, but such patients were more likely to have shock. Our results were relatively consistent with these findings [6, 7], which was reasonable because patients with intra-abdominal or urinary tract infections often had stone pyelonephritis, cholangitis, or cholecystitis, namely, source-controllable infections [9]. On the other hand, mortality associated with intra-abdominal infection varied, which might be due to the wide heterogeneity of intra-abdominal infections such as peritonitis, pancreatitis, cholangitis, and ischemic bowel infections [6]. Mortality rates were the highest among patients with CNS infection and septic shock, but the statistical significance was not clear. Only 23 of 1184 patients with CNS infection were admitted to an ICU, and they were less likely to have shock (*n* = 7 of 23). This might have been underpowered due to the small patient cohort. Otherwise, selection bias might have been involved. Bloodstream and undifferentiated infections might have been also involved in the same discussion as CNS infection. Moreover, the GEE model was used as the main analysis instead of the logistic regression model because the prevalence of site of infection in sepsis and patient’s severity was considered to be clustered by facility, that is, the ordinal logistic regression model could be too efficient, whereas the point estimate from the logistic regression was consistent with that from the GEE model with an independent working correlation matrix. The present results which showed intra-abdominal and CNS infections showed contradictory results about a significance between the GEE model and the logistic regression model. It implied that the severity of intra-abdominal and CNS infections in each ICU was various. Otherwise, it could have needed more samples to show a significance. Previous studies have not found an association between positive blood cultures and mortality outcomes but have associated prognosis more closely with the severity of sepsis rather than the severity of underlying infection [13, 14]. More patients in the present study with infections of the urinary tract (major infection site) also had bacteremia, but the mortality rate was lower than that for other infections. Prognosis might have been associated with the distribution of infection sites in patients with bacteremia in each study.

Septic shock in 63% of the patients in the present study was associated with 28% in-hospital mortality, which was lower than previously published rates [6]. A systematic review identified a crude mortality rate associated with septic shock of 47% [15]. The present study found at least 40% mortality when septic shock was associated with the lungs, CNS, and undifferentiated infections. Although mortality rates are high among patients with septic shock, our findings emphasized the potential contribution of infection sites to mortality. The particular distribution of infection sites and shock might have contributed to the mortality rates in the present study. The contributing influence of infection sites among patients without shock was not clear. Point estimates of odds ratios according to sites of infection were similar despite the presence of shock in most patients, suggesting that the effect of the infection site on mortality was independent of shock. The number of patients without shock may have been insufficient to discern a statistical difference in mortality rates between those with and without shock. Otherwise, mortality rates might not differ according to infection sites among patients without shock. Validation studies with a larger sample size are needed to confirm our findings.

### Possible explanations and implications

We found wide clinical heterogeneity regarding infection sites in patients with severe sepsis and septic shock. This highlights the importance of early and accurate diagnosis of infection sites, with the ability to diagnose warranting equivalent consideration of the skill required to select optimal treatment strategies [8]. By analogy, our findings suggested that because sepsis is not a monolithic disease, the approach should perhaps follow strategies more commonly found in oncology [16]. For example, cancer is uniform in the sense of dysregulated cell growth with distant invasion potential [17], but cancer treatment is now increasingly based on targeted cell receptors and is rapidly moving away from a chemotherapy-for-all paradigm [8]. The present findings imply that increased focus on specific organ-system presentation, namely, “sepsis at the infection source,” with appropriate subsequent risk stratification and diagnosis could be a reasonable strategy. Our results might contribute to similar profiling of infection sites, which we believe will be an important frontier of future sepsis research [8]. Moreover, differences of outcome according to the source infection are important to be considered in future randomized control trials in sepsis and septic shock.

### Limitations

Several limitations of this study warrant discussion. First, the observational design of the study caused difficulties distinguishing causative from correlational relationships. Causal influence requires statistical evaluation, but clinical implications should also be considered. Second, we based our profile classification on suspected sites of infection at the time of admission, rather than on definitive sites of infection. Some patients might have been misdiagnosed, and our results suggest that the consequences of this impact patient outcomes. However, most infection sites were clarified from culture findings (Additional file 2: Table S2). Third, we consolidated 11 initial sites of infection into seven. This might have generated some bias but avoided overcomplexity. Fourth, we enrolled fewer patients than expected during consecutive sampling. The reasons for this were as follows. The prevalence of sepsis is relatively lower in tertiary care centers in Japan (< 5% in the 2010–2011 sepsis registry [18]). Some institutions became involved in the FORECAST study well after it began. Other institutions may have obtained convenience samples even though we had planned consecutive enrolment in the FORECAST study. Fifth, although our data were derived from a large database compiled from 59 ICU, all patients were diagnosed and treated in Japan. Therefore, epidemiological patterns of infection sites and microorganisms might be localized. We studied only patients who were admitted to ICU. Sixth, patients with CNS or soft tissue infections were more likely to be admitted to wards instead of ICU because they were less likely to have shock. Seventh, SSCG 2016 has provided updated definitions and clinical criteria for sepsis (sepsis-3) [2, 15, 19]. Nonetheless, application to the clinical settings would not be difficult due to the large overlap in sepsis-3 and sepsis-2 definitions. Finally, we do not have information about the amount of time that elapsed between the initiation of sepsis and ICU admission, and this might have been associated with outcomes. We also do not have information about source control. However, we believe that all the patients received appropriate source control because all participating institutions are nationally certified emergency centers.

## Conclusions

We found that in-hospital mortality and the clinical features of patients with severe sepsis and septic shock were heterogeneous according to sites of infection.

## Additional files

Additional file 1: **Table S1.** Characteristics and in-hospital mortality of patients with severe sepsis according to 11 sites of infection (*n* = 1184). Features and in-hospital mortality of patients with severe sepsis according to 11 infection sites. (DOCX 27 kb)

Additional file 2: **Table S2.** Characteristics of patients with sepsis according to seven major infection sites (*n* = 1184). Additional features of patients with sepsis according to seven major sites of infection. (DOCX 29 kb)

Additional file 3: **Table S3.** Initial antibiotic treatment administered to patients with severe sepsis (*n* = 1140). (DOCX 28 kb)

## Abbreviations

ADL: Activities of daily living
APACHE II: Acute physiology and chronic health evaluation II
BMI: Body mass index
CCI: Charlson comorbidity index
CI: Confidence interval
CNS: Central nervous system
ED: Emergency department
FORECAST: Focused Outcomes Research in Emergency Care in Acute Respiratory Distress Syndrome, Sepsis and Trauma
GEE: Generalized estimating equation
ICU: Intensive care unit(s)
IQR: Interquartile range
PIPC/TAZ: Piperacillin/tazobactam
SIRS: Systemic inflammatory response syndrome
SOFA: Sepsis-related organ failure assessment
SSCG: Surviving Sepsis Campaign Guidelines
VCM: Vancomycin
