# Supplementary material for: Variations in infection sites and mortality rates among patients in intensive care units with severe sepsis and septic shock in Japan
Source: J Intensive Care. 2019 May 3;7:28. doi: 10.1186/s40560-019-0383-3 (PMC6500015; doi:10.1186/s40560-019-0383-3)
Supplement: Supplementary file 1 — Table S1. Characteristics and in-hospital mortality of patients with severe sepsis according to 11 sites of infection (n = 1184). Features and in-hospital mortality of patients with severe sepsis according to 11 infection sites. (DOCX 27 kb) [file 40560_2019_383_MOESM1_ESM.docx]

| Table S1. Characteristics and in-hospital mortality among patients with severe sepsis according to 11 infection sites (n = 1,184). | | | | | | | | | | | |
| --- | --- | --- | --- | --- | --- | --- | --- | --- | --- | --- | --- |
|  | Suspected infection sites | | | | | | | | | | |
|  | Lung | Intra-abdomen | Urinary tract | Soft tissue | Wound | Osteo-articular | Endocardium | Catheter related | Implant device related | CNS | Undifferentiated |
| Characteristics | 367 (31.0) | 311 (26.3) | 218 (18.4) | 117 (9.9) | 12 (1.0) | 21 (1.8) | 16 (1.4) | 22 (1.9) | 8 (0.7) | 23 (1.9) | 69 (5.8) |
| Septic shock | 197 (53.7) | 226 (72.7) | 153 (70.2) | 68 (58.1) | 7 (58.3) | 11 (52.4) | 10 (62.5) | 16 (72.7) | 4 (50.0) | 7 (30.4) | 46 (66.7) |
| APACHE II score | 25 (18-31) | 21 (16-28) | 22 (17-27) | 22 (16-29) | 20 (13-30) | 17 (14-22) | 27 (18-34) | 23 (18-31) | 23 (11-34) | 27 (20-33) | 26 (18-34) |
| SOFA score | 9 (5-12) | 9 (6-11) | 9 (6-11) | 8 (5-12) | 7 (5-11) | 5 (4-9) | 9 (7-12) | 10 (8-12) | 7 (3-8) | 9 (7-11) | 10 (7-13) |
| Positive blood culture | 133 (36.4) | 154 (49.8) | 160 (73.7) | 67 (57.3) | 8 (66.7) | 17 (81.0) | 13 (81.3) | 20 (90.9) | 7 (87.5) | 16 (72.7) | 41 (60.3) |
| In-hospital mortality  All | 106 (29.7) | 59 (20.0) | 26 (11.9) | 26 (22.8) | 3 (27.3) | 4 (20.0) | 7 (43.8) | 4 (18.2) | 1 (12.5) | 10 (47.6) | 23 (34.8) |
| Shock (n = 745) | 72 (37.9) | 51 (24.2) | 20 (13.1) | 20 (29.9) | 3 (42.9) | 3 (30.0) | 3 (30.0) | 3 (18.8) | 1 (25.0) | 4 (57.1) | 20 (46.5) |
| No shock (n = 439) | 34 (20.4) | 8 (9.5) | 6 (9.2) | 6 (12.8) | 0 (0) | 1 (10.0) | 4 (66.7) | 1 (16.7) | 0 (0) | 6 (42.9) | 3 (13.0) |
| Reported counts (proportions) for categorical and median (interquartile range) for continuous variables. | | | | | | | | | | | |
| Missing data: in-hospital mortality, n = 36. | | | | | | | | | | | |
| APACHE, acute physiology and chronic health evaluation; CNS, central nerve system; SOFA, sequential organ failure assessment. | | | | | | | | | | | |
