# Supplementary material for: Variations in infection sites and mortality rates among patients in intensive care units with severe sepsis and septic shock in Japan
Source: J Intensive Care. 2019 May 3;7:28. doi: 10.1186/s40560-019-0383-3 (PMC6500015; doi:10.1186/s40560-019-0383-3)
Supplement: Supplementary file 2 — Table S2. Characteristics of patients with sepsis according to seven major infection sites (n = 1184). Additional features of patients with sepsis according to seven major sites of infection. (DOCX 29 kb) [file 40560_2019_383_MOESM2_ESM.docx]

| Table S2. Characteristics among patients with sepsis according to 7 major infection sites (n = 1,184). | | | | | | | | | |
| --- | --- | --- | --- | --- | --- | --- | --- | --- | --- |
|  |  |  | Suspected infection sites | | | | | | |
|  |  |  | Lungs | Intra-abdomen | Urinary tract | Soft tissue | Bloodstream | CNS | Undifferentiated |
| Characteristics |  |  | 367 (31.0) | 311 (26.3) | 218 (18.4) | 129 (10.9) | 67 (5.7) | 23 (1.9) | 69 (5.8) |
| qSOFA score | RR ≥ 22 |  | 292 (79.8) | 217 (70.0) | 166 (76.1) | 84 (66.1) | 47 (72.3) | 16 (72.7) | 43 (63.2) |
|  | SBP ≤ 100 |  | 162 (45.0) | 174 (57.6) | 141 (66.2) | 65 (52.0) | 38 (56.7) | 5 (21.7) | 35 (52.2) |
|  | GCS < 15 |  | 268 (73.8) | 186 (60.0) | 142 (65.1) | 71 (55.5) | 46 (68.7) | 22 (95.7) | 43 (64.2) |
| Blood culture | Gram-negative | E. coli | 12 (3.3) | 53 (17.0) | 103 (47.2) | 6 (4.7) | 3 (4.5) | 0 (0) | 5 (7.2) |
|  |  | Klebsiella | 12 (3.3) | 40 (12.9) | 16 (7.3) | 1 (0.8) | 1 (1.5) | 0 (0) | 2 (2.9) |
|  |  | Pseudomonas | 4 (1.1) | 7 (2.3) | 2 (0.9) | 3 (2.3) | 0 (0) | 0 (0) | 0 (0) |
|  | Gram-positive | Staphylococci | 24 (6.5) | 12 (3.9) | 6 (2.8) | 11 (8.5) | 24 (35.8) | 5 (21.7) | 9 (13.0) |
|  |  | Streptococci | 29 (7.9) | 11 (3.5) | 3 (1.4) | 35 (27.1) | 11 (16.4) | 8 (34.8) | 12 (17.9) |
|  |  | MRSA | 4 (1.1) | 2 (0.6) | 1 (0.5) | 5 (3.9) | 3 (4.5) | 1 (4.3) | 0 (0) |
|  |  | Enterococcus | 3 (0.8) | 11 (3.5) | 9 (4.1) | 1 (0.8) | 1 (1.5) | 0 (0) | 0 (0) |
|  |  | Anaerobic | 4 (1.1) | 11 (3.5) | 3 (1.4) | 2 (1.6) | 1 (1.5) | 0 (0) | 3 (4.3) |
|  | Fungi |  | 4 (1.1) | 3 (1.0) | 0 (0) | 1 (0.8) | 4 (6.0) | 1 (4.3) | 1 (1.4) |
| Organ dysfunction on arrival | Hypotension |  | 172 (46.9) | 184 (59.2) | 144 (66.1) | 67 (51.9) | 42 (62.7) | 6 (26.1) | 41 (59.4) |
|  | Hyperlactatemia (>2 mmol/L) | | 228 (62.1) | 230 (74.0) | 155 (71.1) | 87 (67.4) | 37 (55.2) | 16 (69.6) | 44 (63.8) |
|  | Acute kidney injury (Cre >2 mg/dL) | | 122 (33.2) | 113 (36.3) | 96 (44.0) | 54 (41.9) | 29 (43.3) | 8 (34.8) | 33 (47.8) |
|  | Acute lung injury |  | 254 (69.2) | 78 (25.1) | 41 (18.8) | 37 (28.7) | 11 (16.4) | 4 (17.4) | 17 (24.6) |
|  | Hyperbilirubinemia (>2.0 mg/dL) | | 40 (10.9) | 84 (27.0) | 21 (9.6) | 26 (20.2) | 14 (20.9) | 0 (0) | 19 (27.5) |
|  | Thrombocytopenia (<100,000/μL) | | 52 (14.2) | 85 (27.3) | 86 (39.4) | 48 (37.2) | 26 (38.8) | 10 (43.5) | 38 (55.1) |
|  | Coagulopathy (INR >1.5) | | 47 (12.8) | 75 (24.1) | 40 (18.3) | 25 (19.4) | 17 (25.4) | 1 (4.3) | 20 (29.0) |
| Reported counts (proportions) for categorical and median (interquartile range) for continuous variables. | | | | | | | | | |
| Missing data: APACHE II scores, n =162; qSOFA RR, n = 8; SBP, n = 27; GCS, n = 8. | | | | | | | | | |
| APACHE, acute physiology and chronic health evaluation; GCS, Glasgow coma scale; CNS, central nervous system; RR, respiratory rate; SBP, systolic blood pressure. | | | | | | | | | |
