# Supplementary material for: Variations in infection sites and mortality rates among patients in intensive care units with severe sepsis and septic shock in Japan
Source: J Intensive Care. 2019 May 3;7:28. doi: 10.1186/s40560-019-0383-3 (PMC6500015; doi:10.1186/s40560-019-0383-3)
Supplement: Supplementary file 3 — Table S3. Initial antibiotic treatment administered to patients with severe sepsis (n = 1140). (DOCX 28 kb) [file 40560_2019_383_MOESM3_ESM.docx]

| Table S3. Initial antibiotic use among patients with severe sepsis (n=1,140) | | | | | | | | | |
| --- | --- | --- | --- | --- | --- | --- | --- | --- | --- |
|  |  |  | Infection sites | | | | | | |
|  |  | All | Lung | Intra-abdomen | Urinary tract | Soft tissue | Bloodstream | CNS | Undifferentiated |
|  |  | 1,140 | 349 (30.6) | 297 (26.1) | 213 (18.7) | 124 (10.9) | 67 (5.9) | 22 (1.9) | 64 (5.6) |
| Antibiotics |  |  |  |  |  |  |  |  |  |
| Penicillin derivatives (PCG, ABPC, ABPC/MCIPC) |  | 30 (2.6) | 4 (1.1) | 4 (1.3) | 1 (0.5) | 11 (8.5) | 1 (1.5) | 5 (22.7) | 4 (5.3) |
| Ampicillin/sulbactam |  | 78 (6.8) | 44 (12.5) | 11 (3.7) | 4 (1.9) | 7 (5.6) | 5 (7.5) | 2 (9.1) | 5 (7.8) |
| PIPC/TAZ |  | 238 (20.9) | 85 (24.1) | 59 (19.9) | 52 (24.4) | 20 (16.1) | 16 (23.9) | 0 (0) | 6 (9) |
| Sulbactam/cefoperazone |  | 11 (1.0) | 1 (0.3) | 10 (3.4) | 0 (0) | 0 (0) | 0 (0) | 0 (0) | 0 (9.4) |
| First generation cephalosporin |  | 28 (2.5) | 4 (1.1) | 3 (1.0) | 1 (0.5) | 13 (10.5) | 5 (7.5) | 0 (0) | 2 (3.1) |
| Second generation cephalosporin (CTM, CMZ, FMOX) |  | 32 (2.8) | 1 (0.3) | 24 (8.1) | 3 (1.4) | 0 (0) | 1 (1.5) | 0 (0) | 3 (4.7) |
| Third generation cephalosporin (CTX, CPZ, CTRX) |  | 99 (8.7) | 41 (11.6) | 4 (1.3) | 26 (12.2) | 6 (4.8) | 7 (10.4) | 9 (40.9) | 6 (9.4) |
| Third generation cephalosporin against Pseudomonas |  | 4 (0.4) | 0 (0) | 1 (0.3) | 3 (1.4) | 0 (0) | 0 (0) | 0 (0) | 0 (0) |
| Fourth generation cephalosporin against Pseudomonas |  | 26 (2.3) | 9 (2.5) | 6 (2.0) | 2 (0.9) | 3 (2.4) | 1 (1.5) | 3 (13.6) | 2 (3.1) |
| Carbapenem |  | 627 (55.0) | 159 (45.0) | 182 (61.3) | 127 (59.6) | 79 (63.7) | 30 (44.8) | 10 (45.5) | 40 (62.5) |
| Aminoglycoside |  | 8 (0.7) | 3 (0.8) | 0 (0) | 1 (0.5) | 0 (0) | 3 (4.5) | 0 (0) | 1 (1.6) |
| Quinolone |  | 19 (1.7) | 16 (4.5) | 0 (0) | 1 (0.5) | 1 (0.8) | 1 (1.5) | 0 (0) | 0 (0) |
| Tetracycline |  | 7 (0.6) | 5 (1.7) | 0 (0) | 0 (0) | 2 (1.6) | 0 (0) | 0 (0) | 0 (0) |
| Macrolide |  | − | − | − | − | − | − | − | − |
| Metronidazole |  | 12 (1.1) | 0 (0) | 6 (2.0) | 2 (0.9) | 4 (3.2) | 0 (0) | 0 (0) | 0 (0) |
| CLDM |  | 48 (4.2) | 6 (1.6) | 3 (1.0) | 1 (0.5) | 31 (25.0) | 2 (3.0) | 0 (0) | 5 (7.8) |
| Vancomycin |  | 203 (17.8) | 51 (14.4) | 25 (8.4) | 27 (12.7) | 33 (26.6) | 32 (47.8) | 14 (63.6) | 21 (30.4) |
| Other anti–MRSA agents |  | 67 (5.9) | 11 (3.1) | 9 (3.0) | 7 (3.3) | 27 (21.8) | 5 (7.5) | 1 (4.5) | 7 (10.9) |
| Antifungals |  | 50 (4.4) | 21 (5.7) | 9 (3.0) | 5 (2.3) | 5 (4.0) | 6 (9.0) | 1 (4.5) | 3 (4.7) |
| Others |  | 83 (7.3) | 65 (17.7) | 7 (2.4) | 5 (2.3) | 1 (0.8) | 2 (3.0) | 0 (0) | 3 (4.7) |
| Number of antibiotics | 1 | 721 (63.2) | 215 (60.9) | 237 (79.8) | 165 (77.5) | 41 (33.1) | 25 (37.3) | 6 (27.3) | 32 (50.0) |
|  | 2 | 323 (28.3) | 108 (30.6) | 51 (17.2) | 41 (19.2) | 52 (41.9) | 36 (53.7) | 11 (50.0) | 24 (37.5) |
|  | 3 | 80 (7.0) | 25 (7.1) | 8 (2.7) | 7 (3.3) | 28 (22.6) | 5 (7.5) | 3 (13.6) | 4 (6.3) |
|  | ≥4 | 15 (1.3) | 5 (1.4) | 0 (0) | 0 (0) | 3 (2.4) | 1 (1.5) | 2 (9.1) | 4 (6.3) |
| Time to antibiotic use | minutes | 100 (54–185) | 116 (60–191) | 94 (45–201) | 87 (40–141) | 106 (60–189) | 121 (71–240) | 120 (34–197) | 94 (31–175) |
| Reported counts (proportions) for categorical and medians (interquartile ranges) for continuous variables. Missing: antibiotics, n = 44; number of antibiotics, n = 1.  Time to antibiotic use were excluded if it was over 1,440 minutes (n=28)  ABPC, ampicillin; ABPC/MCIPC, ampicillin/cloxacillin; CLDM, clindamycin; CMZ, cefmetazole; CNS, central nervous system; CPZ, cefoperazone; CTM, cefotiam; CTRX, ceftriaxone; CTX, cefotaxime; FMOX, flomoxef; MRSA, methicillin-resistant Staphylococcus aureus; PCG, penicillin G; PIPC/TAZ, tazobactam/piperacillin. Intravenous macrolides were unavailable in Japan during the study period. | | | | | | | | | |
